# Inflammation as a therapeutic target in heart failure with preserved ejection fraction

**DOI:** 10.3389/fcvm.2023.1125687

**Published:** 2023-06-29

**Authors:** Zhen Hui Peh, Adel Dihoum, Dana Hutton, J. Simon C. Arthur, Graham Rena, Faisel Khan, Chim C. Lang, Ify R. Mordi

**Affiliations:** ^1^ School of Medicine, University of Dundee, Ninewells Hospital, Dundee, United Kingdom; ^2^ Division of Molecular and Clinical Medicine, School of Medicine, University of Dundee, Dundee, United Kingdom; ^3^ Division of Cell Signalling and Immunology, School of Life Sciences, University of Dundee, Dundee, United Kingdom; ^4^ Division of Cellular Medicine, School of Medicine, University of Dundee, Dundee, United Kingdom; ^5^ Division of Systems Medicine, School of Medicine, University of Dundee, Dundee, United Kingdom

**Keywords:** inflammation, heart failure, HFpEF, interleukins, cardiometabolic

## Abstract

Heart failure with preserved ejection fraction (HFpEF) accounts for around half of all cases of heart failure and may become the dominant type of heart failure in the near future. Unlike HF with reduced ejection fraction there are few evidence-based treatment strategies available. There is a significant unmet need for new strategies to improve clinical outcomes in HFpEF patients. Inflammation is widely thought to play a key role in HFpEF pathophysiology and may represent a viable treatment target. In this review focusing predominantly on clinical studies, we will summarise the role of inflammation in HFpEF and discuss potential therapeutic strategies targeting inflammation.

## Introduction

Heart failure with preserved ejection fraction (HFpEF) accounts for half of all cases of Heart Failure. Broadly speaking, it is defined as symptoms and signs of HF in the presence of a left ventricular ejection fraction (LVEF) of more than 50%, usually in the presence of a cardiac structural or functional abnormality such as left ventricular hypertrophy, left atrial enlargement or diastolic dysfunction (1). At least in part due to various factors (2) such as increased life expectancy, increasing prevalence of comorbidities such as metabolic syndrome, obesity, diabetes mellitus and greater clinical recognition, the prevalence of HFpEF is increasing such that in future it is likely to be the dominant form of HF worldwide (3).

## Lack of evidence based treatment options for HFpEF: An unmet need

Despite intense research interest in HFpEF, there are few evidence-based treatment options. Randomized trials targeting neurohormonal and sympathetic nervous system using angiotensin converting enzyme inhibitors (ACEi), angiotensin II receptor blockers (ARBs) and beta blockers, which provide clear benefits in HF with reduced ejection fraction (1) (HFrEF), have failed to meet their primary efficacy outcome in HFpEF. The TOPCAT (4) (spironolactone) and PARAGON-HF (5) (sacubitril/valsartan) trials also failed to meet their primary outcomes, though there were perhaps some positive signals in specific groups (for example patients recruited in the Americas (6) in TOPCAT and women or those with mildly reduced ejection fraction (7) in PARAGON-HF). Until recently, the treatment of HFpEF was limited to the used of loop diuretics to relieve congestion and provide symptomatic relief, and optimization of treatment comorbidities such as hypertension and atrial fibrillation.

A recent breakthrough has been the positive outcome trials using sodium-glucose cotransporter 2 (SGLT2) inhibitors in HFpEF. EMPEROR-Preserved (8) and DELIVER (9) demonstrated reductions in cardiovascular death and heart failure hospitalization with empagliflozin and dapagliflozin respectively compared to placebo. Based on these results, SGLT2 inhibitors are likely to play a key role in the management of HFpEF in future (10). Nevertheless, with HFpEF set to the dominant form of HF worldwide yet only having one evidence-based treatment, there is undoubtedly an urgent need for additional effective therapeutic strategies.

## HFpEF: A Complex disease with Complex pathophysiology

One of the key reasons for our inability to develop successful HFpEF treatments is its pathophysiological complexity and our limited understanding of this. This even extends to our definition of HFpEF. The textbook definition of HFpEF as a form of diastolic dysfunction leading to impaired left ventricular filling and reduced cardiac output does not fully capture the complexity of HFpEF. Various definitions of HFpEF have been used in guidelines and for inclusion in clinical trials with different profiles of patients consequently included under the HFpEF umbrella (11). Detailed phenotyping studies have also consistently identified that even when using one definition of HFpEF, patients can be clustered into separate groups that have specific phenotypes and differential clinical outcomes and responses to treatment (12–14).

## Clinical phenotypes in HFpEF

**Figure 1:**
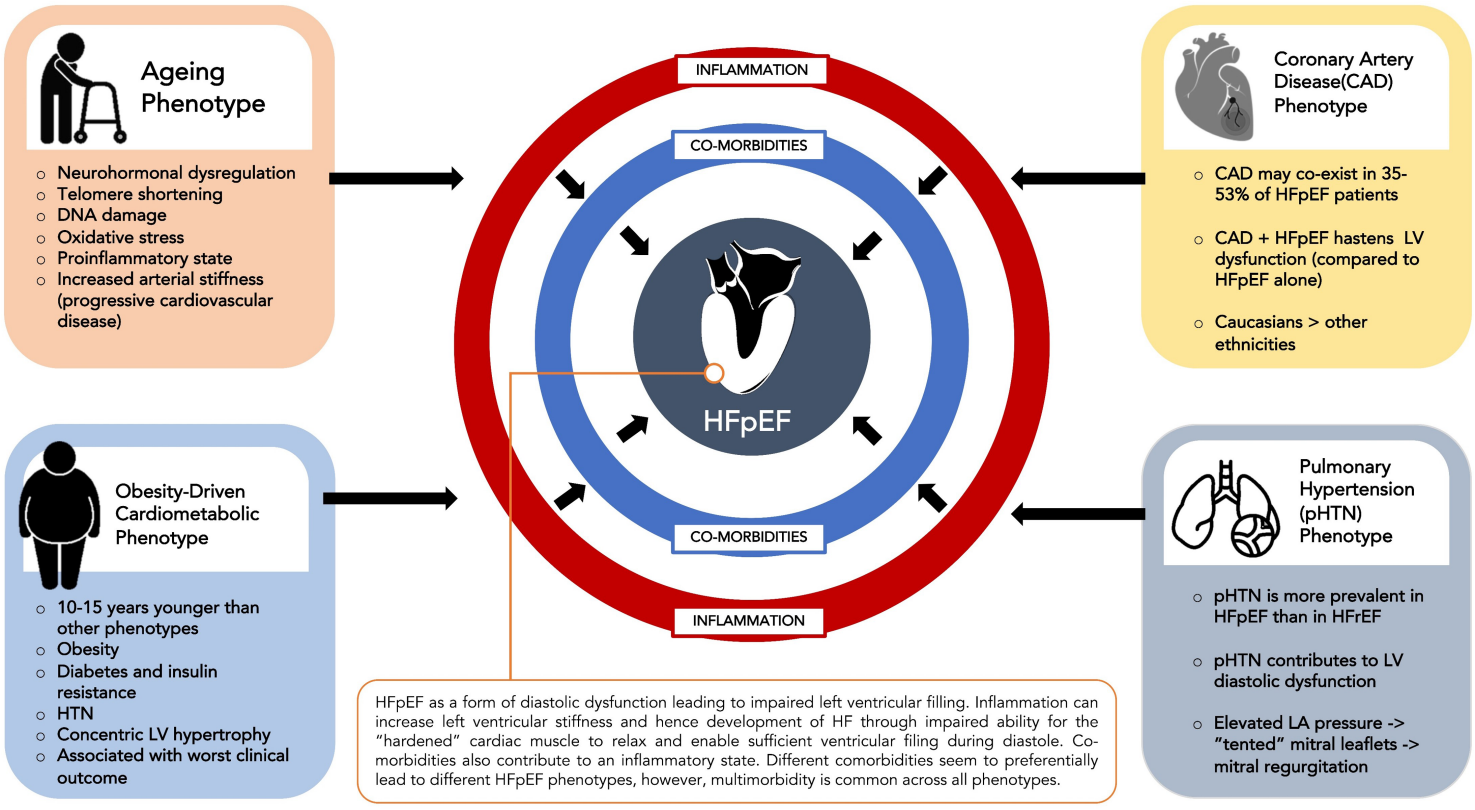
Common HFpEF phenotypes.

HFpEF involves a complex interplay between comorbidities such as hypertension, diabetes mellitus, renal failure, COPD, obesity and atrial fibrillation and cardiac structural and functional abnormalities such as LV hypertrophy, myocardial fibrosis, myocardial stiffness, endothelial dysfunction and oxidative stress (15). Different comorbidities seem to preferentially lead to different phenotypes of HFpEF although multimorbidity is common across phenotypes. The four commonest HFpEF clinical phenotypes (16) are an ageing phenotype, pulmonary hypertension phenotype, coronary artery disease phenotype and the obesity-driven cardiometabolic phenotype (Figure 1).

The cardiometabolic phenotype has been most concisely identified across various HFpEF cohorts. Patients with this phenotype usually have a higher prevalence of comorbidities such as hypertension, obesity, diabetes and insulin resistance and are usually 10–15 years younger (17) than patients diagnosed with other phenotypes of HFpEF. In TOPCAT, this phenotype was found to have more prevalent cardiac structural abnormalities such as concentric LVH and to have worse clinical outcomes compared to other phenotypes (18). This cardiometabolic HFpEF phenotype is consistently identified in HFpEF cohorts (12, 13, 19).

Recently, the term “metainflammation” has been used to describe the association between metabolic stress caused by conditions such as diabetes, obesity, insulin resistance and non-alcoholic fatty liver disease and chronic inflammation (20). This chronic inflammatory state can predispose to adverse cardiac remodeling such as left ventricular hypertrophy that can eventually lead to HFpEF (21). In the RELAX trial, the presence of an increased number of comorbidities such as obesity, diabetes, anaemia and chronic kidney disease in HFpEF patients was associated with higher C-reactive protein (CRP) levels (22). Further demonstrating the association between adiposity, inflammation and HFpEF, visceral adipose tissue (VAT) has been reported as an independent risk factor for hospitalization and mortality in HFpEF (21, 23), particularly in females (24). This is thought to be, at least in part, due to proinflammatory cytokines secreted by VAT that contribute to endothelial dysfunction and reduced vascular compliance (23) and subsequent concentric LV remodeling, reduced left ventricular compliance.

Regardless of the underlying phenotype, what has been consistently identified throughout these studies is that inflammation is highly prevalent in HFpEF and is associated with worse symptoms and prognosis (25). Over half of the patients in a recent HFpEF trial (RELAX) had an elevated C-reactive protein (22). Studies comparing HFpEF and HFrEF cohorts have also consistently identified that inflammation appears to be more prevalent in HFpEF compared to HFrEF, perhaps suggesting a different pathophysiology. In the BIOSTAT-CHF study, a biomarker analysis identified that there were specific biological pathways related to inflammation that were specific to HFpEF, in contrast to HFrEF (26). This finding was validated in a second cohort and replicates an earlier analysis from TIME-CHF that identified that biomarkers related to inflammation such as IL-6 and hsCRP were significantly upregulated in HFpEF compared to HFrEF (27). Levels of biomarkers such as IL-6 and CRP have also been associated with increased risk of incident HFpEF in community patients (28, 29).

As well inflammation being upregulated in HFpEF compared to HFrEF, inflammation is also associated with worse cardiac structural and functional abnormalities in HFpEF patients. A recent proteomic study of 228 HFpEF patients studied 47 proteins involved in inflammatory pathways and found that systemic inflammation was associated with an increased number of comorbidities, diastolic dysfunction and right ventricular dysfunction. Inflammation was also upregulated compared to controls (30). This was similar to other biomarker studies that have identified inflammation mediated by comorbidities as a key component of HFpEF (25).

Upregulation of inflammation in HFpEF not only leads to worse cardiac structure and function but is also associated with worse prognosis, demonstrated using various inflammatory biomarkers. Several studies have reported an independent association between elevated C-reactive protein and increased risk of death or HF hospitalisation in HFpEF patients, including after adjustment for natriuretic peptide levels (22, 31–33). An elevated neutrophil to lymphocyte ratio, a simple measure of systemic inflammation, is also associated with adverse outcome in HFpEF (34). High levels of interleukin-6 are common in HFpEF (35) and were associated with adverse outcome. In the KaRen study of 86 HFpEF patients, higher levels of inflammation were associated with worse NYHA class (25). Elevated levels of inflammatory markers including GDF-15 and TNF receptor 1 have also been found to be independently significantly associated with a composite outcome of HF hospitalisation or all-cause mortality.

In summary, there is an abundance of evidence (albeit predominantly observational) suggesting that inflammation could play a key role in the pathophysiology and clinical outcomes of HFpEF.

## The innate immune system in HFpEF

The innate immune system has been proposed as a key mediator of inflammation in heart failure. Oxidative stress and hypertension cause the recruitment of inflammatory leukocytes, the most notably being neutrophils which is the most abundant leukocytes in circulation. In response to endothelial cells damaged by systemic inflammation, neutrophils secrete MPO. MPO also reduces NO availability (36). Both of these exaggerate the inflammatory response. When neutrophils die, they shed IL-6 receptors that triggers surrounding endothelial cells to recruit more monocytes and macrophage (37), further amplifying the inflammatory response.

**Figure 2:**
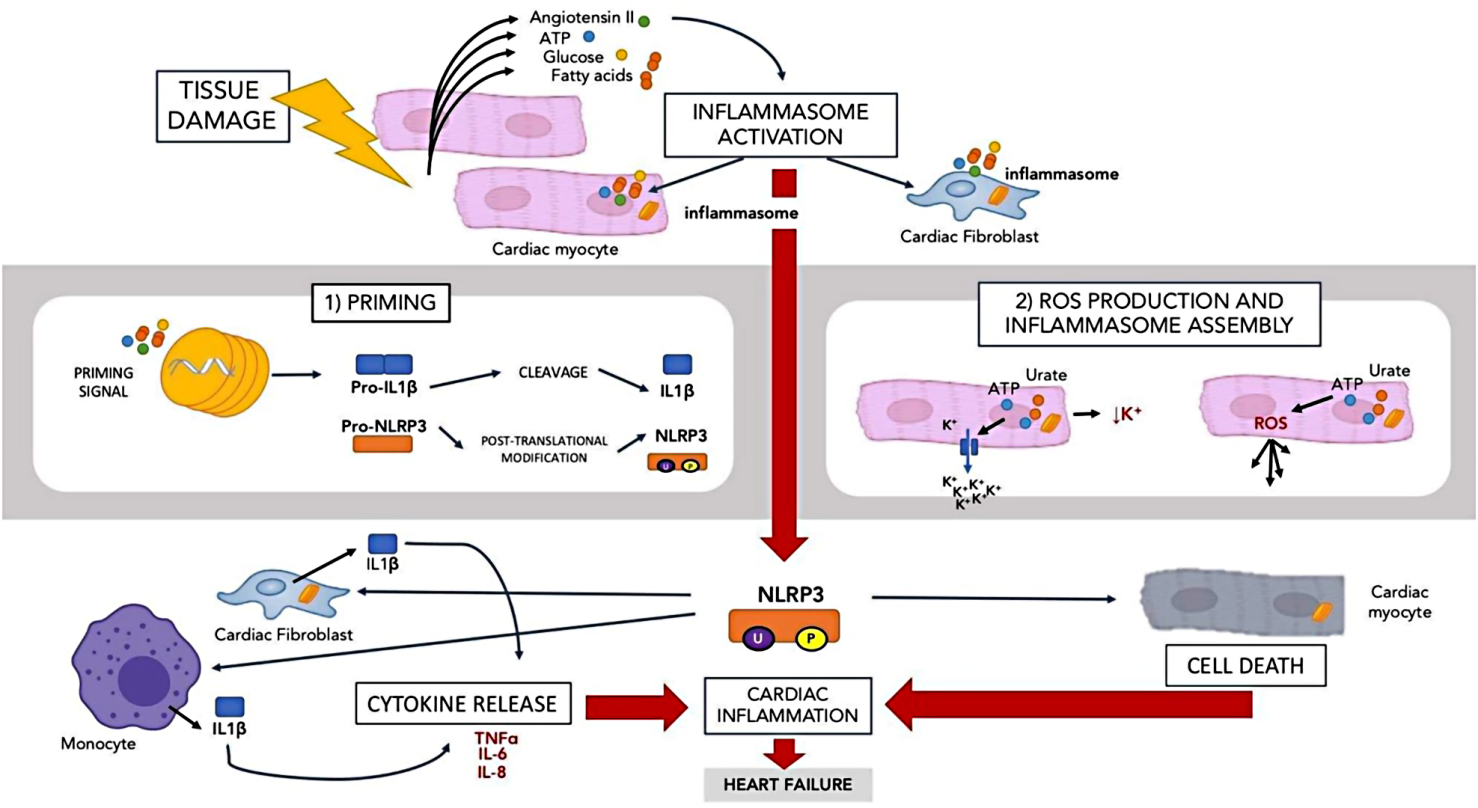
The innate immune system and HFpEF.

In the context of the innate immune system, inflammasomes are also activated by tissue damage. The Nod-like receptor pyrin domain containing 3 (NLRP3) inflammasome is the best described inflammasome relating to the heart (38). NLRP3 is found in the cardiomyocytes and cardiac fibroblasts (39). The NLRP3 inflammasome is summarised in Figure 2.

When tissue is damaged, molecules such as adenosine triphosphate, angiotensin II, fatty acids and glucose are released. The presence of these molecules activates NLRP3 in a 2-step manner (40). In the first step, there is a priming signal that leads to the transcription of IL-1 beta and NLRP3 precursors. NLRP3 precursors then undergo post translation modification via phosphorylation and ubiquitination to remain in a stable form until the second signal is activated. In the second step, the second activating signal such as ATP and urate crystal reduces in intracellular potassium and increase ROS while assembling the inflammasome. A large amount of IL-1beta is also produced by inflammatory cells such as macrophages and cardiac fibroblast (41) in response to NLRP3 activation. IL-1 beta then initiates several downstream inflammatory pathways by inducing other cytokines such as TNF, IL-6 and IL-8 (42). Activation of NLRP3 in the cardiomyocytes leads to cell death rather than IL-1beta release (43). NLRP3 activation causes IL-1 beta release by cardiac fibroblasts and cardiomyocytes death. Inhibition of NLRP3 has been shown to prevent inflammasome activation and cardiac cell death, hence reducing damage (43) and therefore may represent a potential treatment target in HFpEF.

## The adaptive immune system in HFpEF

Although much research has been focused on the innate immune system in HF, there is increasing recognition of the role of the adaptive immune system in HF pathophysiology. It is hypothesised that the adaptive immune system may play and important role in the remodeling process in response to chronic myocardial injury (44, 45). Endomyocardial biopsies of HFpEF patients show the presence of inflammatory cells and CD3+, CD11a+ and CD45+ cells, indicating underlying cardiac inflammation involving the adaptive immune system (46). Histopathological samples from HFpEF patients also demonstrate the presence of increased expression of adhesion molecules that control extravasation of T cells (46, 47). T cells may also play a role in cardiac ageing including diastolic dysfunction that is a precursor to HFpEF (48). Many of the comorbidities that are often present in HFpEF are associated with increases in circulating T cells. The metabolic changes seen in HFpEF promote pro-inflammatory T cell differentiation and lead to T cell recruitment in the heart and vasculature (49, 50). The presence of B cells may worsen cardiac injury via a few mechanisms such as the activation of T cells or the innate immune system, production of inflammatory cytokines such as IL-6, IL-1β and TNF-α or recruitment of the innate immune cells through chemokine production (51). Each of these mechanisms then further worsens the inflammatory state in the heart.

## Role of nitrosative stress in the mechanisms behind inflammatory cardiac damage

There is a large amount of evidence suggesting the role obesity and metabolic stress play in the inflammatory state of HFpEF, particularly relating to inflammation-dependent oxidative and nitrosative stress (52–56). The presence of these comorbidities increases reactive oxidative species (ROS) in the cardiac endothelial cells which further contributes to the decrease in endothelial nitric oxide (NO) (56). This results in a reduction in soluble guanylate cyclase (sGC), cyclic guanosine monophosphate (cGMP)) and cGMP protein kinase activity in cardiomyocytes which exerts a cardioprotective (57, 58) effect. The result of this is the remodeling of the left ventricle, impairing relaxation. An observation made in HFpEF is the accumulation of misfolded protein in the myocardium (59), suggesting that the unfolded protein response that mitigates stress in protein quality control is downregulated (60). In HFpEF, the pro-inflammatory molecule inducible nitric oxide synthase (iNOS) is upregulated as seen in both murine models and clinical HFpEF (20, 55).

## Clinical strategies to target inflammation in HFpEF

With the burgeoning observational evidence and mechanistic studies supporting the role of inflammation in HFpEF, there has been intense interest in clinical trials of anti-inflammatory therapies in HFpEF. In this section we will summarise previously tested and potential future strategies targeting inflammation in HFpEF.

## Established HF therapies

### RAAS blockers

The renin-angiotensin-aldosterone system (RAAS) is central in the development of heart failure (61). When the RAAS is activated there is a reduction in blood flow to the kidney and through a cascade of different neurohormones and eventually aldosterone acts on multiple system to increase cardiac output. While initially compensatory, eventually, it acts to exacerbate heart failure. It is also involved in the inflammatory pathways of heart failure. Aldosterone encourages the expansion of adipose tissue and its transition to a proinflammatory state (62) which increases the release of proinflammatory cytokines (63). Other than mediating microvascular dysfunction and fibrosis (64), aldosterone is also found to contribute to the development of experiment HFpEF (65). Levels of aldosterone correlate to LV hypertrophy that is frequently found in HFpEF (66) and high level of aldosterone usually precede the development of metabolic syndrome (67) that also contributes to systemic inflammation. In hypertensive patients RAAS blockers do reduce inflammation (68). Hence it would seem intuitive that inhibition of the RAAS would help in the treatment of HF.

Unlike in HFrEF however, RAAS blockade in HFpEF does not improve clinical outcomes to anywhere near the same extent (69). In the TOPCAT (4) trial, spironolactone did not significantly reduce the incidence of mortality and hospitalization rates, though there were some subgroups of patients who may have benefited. Nevertheless, in a post-hoc analysis of the trial spironolactone did not cause any reduction hsCRP compared to placebo (70). Sacubitril/valsartan may be beneficial in HF with mildly reduced EF (71) and may reduce systemic inflammation in HFrEF (72), however there is no evidence as to whether this is also the case in HFpEF. Given the overall, at best neutral results of RAAS inhibitors in HFpEF it is unlikely that any anti-inflammatory effect exerted would improve clinical outcomes.

### Beta-blockers

Beta-blockers reverse the neurohormonal effects of the sympathetic nervous system, improving symptoms and outcomes in HFrEF (73, 74). Beta-blockers also appear to reduce inflammation in HF. Hs-CRP concentration and oxidative stress in patient with chronic heart failure has been shown to be reduced by beta blocker such as bisoprolol and carvedilol (4), and in dilated cardiomyopathy, use of beta blockers is associated with a reduction in inflammatory markers such as IL-10 and TNF alpha (75).

The evidence for beta-blockers in HFpEF is weaker however (76) though there may be some reductions in mortality (77). Beta-blockers are often prescribed for other indications in patients with HFpEF such as atrial fibrillation and coronary artery disease (78). Again however, there are very few studies investigating inflammation in response to beta-blockers in HFpEF.

## SGLT2 inhibitors

Sodium-Glucose co-Transporter 2 (SGLT2) Inhibitors have recently become an established part of HF care, following their initial development as type 2 diabetes treatments. They act by blocking SGLT2, a protein in the kidney that promotes the reabsorption of glomerular filtrated glucose back into the systemic circulation, contributing to about 90% of glucose reabsorption (79), causing a significant diuresis that is beneficial in HF (80). SGLT2 inhibitors cause significant reductions in HF hospitalisations and mortality in HFrEF patients with or without type 2 diabetes (81–83), and also improve outcomes in HFpEF (8, 9), meaning that for the first time we have a treatment for HFpEF that improves clinical outcomes.

The mechanisms of benefit of SGLT2 inhibitors in HF are still unclear and are a subject of intense ongoing research interest (84–86). SGLT2 inhibitors also appear to help in lowering systemic inflammation. A systematic review and meta-analysis of studies (87) in rodents showed that the use of SGLT2 inhibitors resulted in a decrease in IL-6, CRP, TNF alpha and MCP-1. SGLT2 inhibitors were also shown to significantly suppress NLRP3 inflammasome activation and in turn IL-1 beta secretion in human macrophages (88). SGLT2 inhibitors reduce epicardial fat, a source of pro-inflammatory cytokines, independently of their glycaemic effects (89, 90). In a recent trial of dapagliflozin vs. placebo in diabetic individuals with LVH, 12 months of treatment with dapagliflozin caused a significant reduction in hsCRP (91). Though is likely that the mechanisms of benefit of SGLT2 inhibitors in HF are likely to be multi-factorial, reductions in systemic inflammation may well be contributory.

## Exercise

Exercise Training can be a potential means of reducing systemic inflammation. Reduced exercise capacity is a significant part of the problem in patients with HFpEF, leading to reduced quality of life and worse prognosis (92). The current ESC guidelines (1) recommend exercise to improve exercise capacity, QoL and reduce HF hospitalization. The bulk of the evidence for exercise training applies to HFrEF, though there may be some benefit in HFpEF patients (93, 94).

Regular exercise helps to exert antioxidant and anti-inflammatory effects by targeting the cardiovascular system, adipose tissues and immune system (95). Upon muscle contraction, IL-6 is released by the skeletal muscle into the bloodstream. This release of IL-6 does not come with a similar release of TNF-alpha and IL-1beta, resulting in an overall anti-inflammatory effect (96). IL-6 also increases anti-inflammatory markers such as IL-1 receptor antagonist and IL-10 (97) which further exerts an inhibitory effect on TNF alpha and IL-1beta (98). Adipose tissue which serves as a source for TNF-alpha is also reduced by regular exercise (99). Lastly, NLRP3 inflammasome activation is also inhibited (100). eNOS is also upregulated by exercise resulting in increased bioavailability of NO leading to improved vascular function and reduced oxidative stress (101).

Few clinical studies have as yet evaluated the effects of exercise training on markers of inflammation. It is possible that systemic inflammation may also modify the effects of exercise in HFrEF (102). There are ongoing large studies of exercise in HFpEF (103) that will shed more light on the effects of exercise and could provide an avenue for exploring mechanisms of action.

## Systemic anti-inflammatory therapy

### Methotrexate

Methotrexate is an anti-inflammatory commonly used in conditions such as rheumatoid arthritis (RA). Methotrexate use was associated with a reduction in cardiovascular events in patients with rheumatological conditions (104). A recent retrospective study (105) of 9,889 patients with RA and matched controls from the Vanderbilt University Medical center electronic health record, treatment with methotrexate was associated with lower risk of incident HFpEF. There may be a role for methotrexate in the management of inflammation in HFpEF, particularly in patients with a high inflammatory profile.

There have been no specific trials of methotrexate in HFpEF as yet. In the CIRT (106) (Cardiovascular inflammation Reduction Trial) trial, in which individuals with a history of MI or coronary artery disease who had either type 2 diabetes or metabolic syndrome were randomized to low-dose methotrexate (up to 20 mg daily) or placebo. Around 13% of participants had a history of HF. After a median of follow up of 2.3 years methotrexate did not reduce levels of IL6, Il1beta and CRP, nor did it reduce incidence of the primary endpoint of cardiovascular death, myocardial infarction or stroke. In a small randomized trial in 71 HFrEF patients 12 weeks of methotrexate treatment did cause a reduction in inflammatory markers and improved NYHA class, quality of life and increased 6-minute walk distance, without any change in LV ejection fraction.

### Colchicine

Colchicine is an anti-inflammatory agent more commonly used to treat conditions such as gout, pericarditis and Behcet's syndrome. It exerts its anti-inflammatory effect by blocking the activation of NLRP3 inflammasome which in turn reduces the production of IL-1 beta and IL-18, inhibiting tubulin polymerization and microtubule development and thus impairing neutrophil migration. This inhibits IL-1 production by activated neutrophils and down regulates TNF alpha receptors in macrophages and endothelial cells (107).

The concept of using colchicine for chronic cardiovascular disease was tested in the COLCOT (108) trial. In this study of 4,745 individuals post-myocardial infarction randomized to colchicine or placebo there was a 23% relative risk reduction in the primary CV endpoint. Only 2% of the study population had a history of heart failure however. One small trial of colchicine in HFrEF patients found that although colchicine caused a reduction in inflammatory markers (CRP and IL-6) it did not cause any improvements in exercise capacity, symptoms or reductions in HF hospitalisation or mortality (109).

There is no clinical trial data as yet in HFpEF. A recent animal study using a murine hypertensive HFpEF model found that treatment with colchicine results reduced cardiac diastolic dysfunction, oxidative stress and fibrosis and improved exercise capacity (110). The ongoing COLpEF trial (NCT04857931) is an ongoing randomized clinical trial testing the efficacy of colchicine in reducing inflammation and improvement in left ventricular diastolic function and functional status and symptoms of patients with HFpEF and will provide further data on the role of colchicine in HFpEF.

### Statins

In addition to their cholesterol-lowering properties, statins have many pleiotropic effects. These include anti-inflammatory properties—statins reduce CRP levels by 15%–30% (111). They act by inducing eNOS which improves endothelial function, inhibit adhesion molecules such as VCAM-1 and ICAM-1, reduce the effect of NFKB and disrupt T cell activation (111).

Observational studies and post-hoc subgroup analyses had suggested that statins might improve outcomes in HF patients (112–114) however large clinical randomized trials involving statins use in HFrEF failed to show any benefit (115–118). Many HFpEF patients are likely to be prescribed statins (for example for coronary artery disease)—as an example, almost 70% of patients in EMPEROR-Preserved were taking statins at baseline. Given the negative results of statins in HFrEF trials, and the widespread use of statins in HFpEF for other indications, it is unlikely that there will be a randomized trial of statin vs. placebo in HFpEF.

Other potential treatment approaches involve modulating part of the immune system in patients with heart failure to bring about a net anti-inflammatory effect. This includes the use of Prednisolone, Xanthine Oxidase Inhibitor, Pentoxifylline, IVIG or Thalidomide. Table 1 summaries some of the trials involving their use the outcomes from their use. However, most of the trials involve patients with HFrEF and outcomes have been mixed at best. Further research and trials are needed to investigate their relevancy and effect in HFpEF.

**Table 1:** Summary of completed and ongoing clinical trials targeting inflammation in heart failure.

Agent	Trial	LVEF (%)	NYHA Class	Type of Study	Patients (*n*)	Comparison	Main Clinical Outcomes
Methotrexate	METIS (119)	<45%	II–IV	Randomised, double-blinded, placebo-controlled	50	7.5 mg methotrexate, vs. placebo, +5 mg folic acid	Improved NYHA class.
						Duration: 12 weeks	No change in 6 MWT distance.
	Gong et al., 2006 (120)	<45%	II–IV	Randomised, single-blinded, placebo-controlled	71	7.5 mg methotrexate, vs. placebo, once a week	Improvement in NYHA, QoL (SF-36) and 6 MWT distance.
						Duration: 12 weeks	
Colchicine	Deftereos et al., 2014 (109)	<40%	I–III	Randomised, double-blinded, placebo-controlled	279	Colchicine 0.5 mg TDS, vs. placebo	Reduction in inflammatory biomarker levels.
						Duration: 6 months	No change in NYHA or exercise treadmill time.
	COLpEF, NCT04857931	>45%	II–IV	Randomised, double-blinded, placebo-controlled	426	Colchicine 0.5 mg OD, 0.5 mg BD, vs. placebo	Still ongoing.
						Duration: 6 months	Primary outcome is change in hs-CRP.
							Secondary outcomes include change in NT-proBNP, hs-Tn, LV diastolic function and NYHA class.
MPO Inhibition	SATELLITE (121)	>40%	II–IV	Randomised, double-blinded, placebo-controlled	41	2.5 mg AZ4832 OD (escalated to 5 mg after 10 days) vs. placebo	No change in 6 MWT distance, NT-proBNP or QoL (KCCQ).
						Duration: 90 days	
TNFα	ATTACH (122)	<35%	III–IV	Randomised, double-blinded, placebo-controlled	150	Infliximab 5 mg/kg, infliximab 10 mg/kg or placebo at 0, 2 or 6 weeks	No improvement in NYHA class or QoL (MLwHF).
						Duration 28 weeks	More adverse events (death/HF hospitalisation) in high-dose infliximab group.
	RENEWAL (123)	<30%	II–IV	Randomised, double-blinded, placebo-controlled	2,048	RECOVER: SC Etanercept 25 mg weekly or bi-weekly; vs. placebo	No difference in primary composite outcome (death, HF hospitalisation, NYHA class and patient global assessment).
						RENAISSANCE: SC Etanercept 25 mg bi-weekly or trii-weekly; vs. placebo	
						Duration: 24 weeks	
	Deswal et al., 1999 (124)	<35%	III	Randomised, double-blinded, placebo-controlled	18	Escalating dose (1, 4, or 10 mg/m^2^) of etanercept (single IV infusion), vs. placebo	Improvement in QoL (VAS), LVEF, 6 MWT at higher doses.
						Duration: 14 days	
	Bozkurt et al., 2001 (125)	<35%	III–IV	Randomised, double-blinded, placebo-controlled	47	Bi-weekly SC Etanercept 5 mg/m^2^, 12 mg/m^2^, or placebo.	Increase in LVEF in a dose-dependent manner.
						Duration: 3 months	
Interleukin-1	D-HART (126)	> 50%	II–III	Randomised, double-blinded, placebo-controlled	12	Anakinra 100 mg vs. placebo	Improved peak VO_2_.
						Duration: 14 days + additional 14 days of alternate treatment	
	D-HART2 (127)	>50%	II–III	Randomised, double-blinded, placebo-controlled	31	Anakinra 100 mg OD vs. placebo	No improvement in peak VO_2_ and VE/VO_2_ slope.
						Duration: 12 weeks	Reduced NTproBNP and improved QoL.
	REDHART (128)	<50%	II–III	Randomised, double-blinded, placebo-controlled	60	Anakinra 100 mg SC OD for 2 weeks, 12 weeks, vs. placebo	No improvement in peak VO_2_ or VE/VCO_2_ slope at 2 weeks, improved peak VO_2_ at 12 weeks from baseline but no difference between groups.
							No change in LVEF.
							Improvement in QoL in those treated for 12 weeks.
Steroids	COPE- ADHF (129)	<45%	N/A	Randomised, non-blinded	102	IV Dexamethasone 20 mg daily and oral Prednisolone 1 mg/kg daily vs. standard of care	Reduction in cardiovascular death at 30 days.
						Duration: 7 days, then steroid tapered off within 3 days.	Improvement in dyspnoea and physician-assessed clinical status.
	Parrillo et al.,1989 (130)	<35%	N/A	Randomised, controlled, prospective	102	Daily dose of 60 mg of prednisolone for 3 months	Small increase in LVEF after 3 months but not significantly different to control group. The increase was not sustained after 9 months.
Xanthane Oxidase Inhibition	EXACT-HF Study (131)	<40%	II–IV	Randomised, double-blinded, placebo-controlled	253	Allopurinol 300- 600 mg OD, vs. placebo	No significant difference in primary outcome (clinical composite of death, HF hospitalisation, urgent HF visit, medication change orpatient global assessment) compared to placebo.
						Duration: 24 weeks	No change in KCCQ or 6 MWT distance.
	OPT-CHF (132)	<40%	III–IV	Randomised, double-blinded, placebo-controlled	405	Oxypurinol 600 mg OD, vs. placebo	No significant improvement in composite clinical outcome (CV death, HF hospitalisation/worsening HF, medication change, change in NYHA class or patient clinical status).
						Duration: 24 weeks	No change in QoL (MLWHF) or 6 MWT distance.
Statin	GISSI-HF (117)	Any	II–IV	Randomised, double-blinded, placebo-controlled	4,463	Rosuvastatin 10 mg OD, vs. placebo	No effect on primary outcome (death/CV hospitalisation).
	CORONA (116)	<40%	II–IV	Randomised, single-blinded, placebo-controlled	5,011	Rosuvastatin 10 mg ODS daily, vs. placebo	No effect on primary outcome (CV death/non-fatal MI/non-fatal stroke).
							Reduction in all-cause and CV hospitalisation.
	UNIVERSE (115)	<40%	II–IV	Randomised, double-blinded, placebo-controlled	85	Rosuvastatin 10 mg OD for 6 weeks, then 20 mg OD 6 weeks then 40 mg OD for 14 weeks vs. placebo	No significant change in LVEF, BNP or patient global assessment.
						Duration: 26 weeks	
Pentoxifylline	Skudicky et al., 2001 (133)	<40%	II–III	Randomised, double-blinded, placebo-controlled	39	Pentoxifylline 400 mg TDS vs placebo	Improvement in LVEF, NYHA class and exercise time.
						Duration: 6 months	
	Sliwa et al., 1998 (134)	<40% (dilated cardiomyopathy)	II–III	Randomised, double-blinded, placebo-controlled	28	Pentoxifylline 400 mg TDS, vs. placebo	Improvement in LVEF, NYHA class.
						Duration: 6 months	
	Sliwa et al., 2002 (135)	<40% (peripartum cardiomyopathy)	IV	Randomised, double-blinded, placebo-controlled	18	Pentoxifylline 400 mg TDS, vs. placebo	Improvement in LVEF.
	Sliwa et al., 2004 (136)	<40% (ischaemic)	II–III	Randomised, double-blinded, placebo-controlled	38	Pentoxifylline 400 mg TDS, vs placebo.	Improvement in LVEF.
						Duration: 6 months	
	Bahrmann et al., 2004 (137)	<40%	II–III	Randomised, double-blinded, placebo-controlled	47	Pentoxifylline 600 mg BD, vs placebo.	No change in LVEF, NYHA class or symptoms.
						Duration: 6 months	
Immunoglobulins	Gullestad et al., 2001 (138)	<40%	II–III	Randomised, double-blinded, placebo-controlled	40	IVIG vs. placebo	Increase in LVEF.
						Duration: 26 weeks	
	Mcnamara et al., 2001 (139)	<40%	I–IV	Randomised, double-blinded, placebo-controlled	62	2 g/kg IVIG vs. placebo	No significant change in LVEF compared to placebo.
Thalidomide	Gullestad et al., 2005 (140)	<40%	II–III	Randomised, double-blinded, placebo-controlled	56	25 mg QD thalidomide (increasing to 200 mg) vs. placebo	Increase in LVEF, with decrease in LVEDV.
						Duration: 12 weeks	

## Targeted anti-inflammatory therapies

### TNF alpha

As discussed, TNF alpha is one of the more prominent cytokines involved in inflammatory pathway of HF, associated with impaired systolic and diastolic function and adverse cardiac remodeling, and therefore has been well-studied in HF. This was supported by a small early trial that reported that TNF alpha inhibition using etanercept improved LV function at 3 months in HFrEF patients (125). Unfortunately these results were not borne out in large outcome trials. In the RENEWAL trial, etanercept, a TNF alpha receptor inhibitor did not reduce the incidence of primary outcomes of mortality or hospitalization for HF (123), while in ATTACH (122), TNF Inhibitor infliximab actually caused an increased risk of hospitalization and mortality at high doses.

These results may be partly explained by the inhibition of NFKB, a transcription regulator that is activated by TNF alpha and acts as a key effector of it. NFKB has cardioprotective effect such as reducing mitochondrial dysfunction and mitophagy, inhibiting cell death and inducing antioxidant effects (141). With the inhibiting of TNF alpha and consequently NFKB, these beneficial effects may be lost, causing HF to worsen. Furthermore, infliximab has been shown to induce apoptosis and complement mediated cell lysis (141), further contributing to cell death in the failing heart.

These trials have limited enthusiasm for targeting TNF alpha in HF, although they have only included HFrEF patients. Similar studies involving HFpEF have not been performed. There is preliminary data suggesting that anti-inflammatory therapy may be beneficial in certain subgroup of HFpEF. Unlike HFrEF where prevention of cell death in the myocardium is relevant, HFpEF is characterized by myocardial hypertrophy (142). It is conceivable that anti-TNF alpha therapy might have different effects in HFpEF. In an observational study involving the effect of anti-TNF therapy on cardiac function in rheumatoid arthritis (where HFpEF is more prevalent), it was found that anti-TNF alpha therapy was not associated with a worsening of cardiac function and in fact was associated with a 23% decrease in NT-proBNP after 6 months (143). In a Swedish registry on HF in patients with RA, patients treated with corticosteroids had a higher incidence of non-ischemic heart failure compared to patients which used biologics (144). There may still be a role for anti-TNF strategies to be tested in HFpEF.

### Interleukin-1

IL-1 plays an important role in the development of systolic and diastolic dysfunction. In the context of diastolic dysfunction, IL-1 reduces calcium reuptake by sarcoplasmic reticulum through down regulation of phospholamban and SERCA, in turn affecting the initiation of cardiomyocytes relaxation (145, 146).

In the DHART trial, 2 weeks treatment with anakinra, an IL-1 receptor antagonist, reduced systemic inflammation, increased aerobic exercise tolerance and peak VO2 in patients with HFpEF (126).

Unfortunately, in the larger follow-on study DHART2 (127), despite again finding a reduction in CRP, NTproBNP and an improvement in exercise tolerance with anakinra, there was no improvement in the primary endpoint of peak VO2. The discordant results could be at least in part due to the fact that in DHART2, most of the participants were obese which in turns affects their cardiorespiratory fitness regardless of their cardiac function.

Further interest in IL-1 beta blockade as a therapeutic strategy in HF was raised by an analysis of the CANTOS (147) trial. In CANTOS, the anti-IL-1 beta monoclonal antibody canakinumab was studied in patients with a history of MI and evidence of systemic inflammation (measured by elevated hs-CRP). Participants were treated with 150 mg canakinumab or placebo every 3 months with an optimized statin regimen. Canakinumab caused a 15% decreased risk in mortality and non-fatal stroke and MI (147). In a prespecified sub analysis of the trial, canakinumab was found to reduce heart failure-related hospitalization in a dose dependent manner (148). In CANTOS, HFrEF and HFpEF was not discriminated. However, considering that many patients were older and has a history of obesity, diabetes and hypertension, this hypothesis generating result raises the possibility that IL-1 beta blockade with canakinumab might have benefit in patients with HFpEF. Further studies of IL-1 beta blockade in HFpEF are warranted.

### Myeloperoxidase

Myeloperoxidase (MPO) is a heme containing peroxidase that is expressed mainly in neutrophils. They play a role in the development of acute and chronic vascular inflammation which is proposed as an underlying mechanism in the development of HFpEF (149). In the SATELLITE (121) (Safety and Tolerability Study of AZD4831 in Patients with Heart Failure) trail, MPO inhibitor (AZD4831) was investigated. This trial was stopped prematurely after it achieved its original aim of target engagement and satisfactory safety profile. From the trial, it showed a 69% decrease in MPO activity within the 30 days trial period and an increase in exercise capacity and wellness score, highlighting its role as a potential treatment for HFpEF. However, MPO-inhibition is a novel approach in treating HFpEF, hence further studies and trials are needed into to further out understanding and investigate the efficacy of this potential treatment.

## Future research and therapy

There remain few evidence-based treatment options for HFpEF and there is an urgent need for novel therapeutic strategies. As described in Table 1 there are a number of ongoing clinical trials evaluating anti-inflammatory strategies for HFpEF. Given the heterogeneity of HFpEF, a targeted approach with detailed patient phenotyping is likely to be the most fruitful strategy. Using such an approach, patients with high levels of inflammation could be selected for anti-inflammatory therapy and could have more chance of deriving clinical benefit. This could lead to a “precision” approach where a specific cytokine is targeted. An alternative approach would be to use interventional strategies that might have multiple effects, of which an anti-inflammatory action is one. Such strategies include exercise and drugs inducing weight loss (150).

## Conclusions

There is a plethora of observational and trial evidence supporting the role of inflammation as an important part of the pathophysiology of HF. Although the CANTOS trial showed the merit of an anti-inflammatory approach in cardiovascular disease, most other trials of anti-inflammatory therapy in HF have been neutral at best, though these have predominantly been in HFrEF. Very few clinical trials of anti-inflammatory strategies have been conducted in HFpEF populations however, and it is possible that inflammation may play a differential role in HFpEF to HFrEF. It may also be that patients could be selected for trials based on baseline levels of inflammation—those with higher levels of systemic inflammation might be more likely to benefit from anti-inflammatory therapy. Such a strategy needs to be refined as there are many potential inflammatory markers that could be used however we do not know which might translate to clinical benefit. The most widely-used is high-sensitivity C-reactive protein—in the CANTOS trial patients were eligible if their hsCRP level was ≥2 mg/l (147). Optimal levels still need to be defined. Despite the recent breakthrough of SGLT2 inhibitors in treatment of HFpEF, there is a need for further therapeutic options. Inflammation remains a viable treatment target in HFpEF and further well-designed trials are warranted.
